# Pediatric urology in India

**DOI:** 10.4103/0970-1591.42633

**Published:** 2008

**Authors:** V. Raveenthiran, Y. K. Sarin, M. Bajpai

**Affiliations:** Division of Pediatric Surgery, Annamalai University, Tamilnadu, India; 1 Secretary, Pediatric Urology Section Indian Association of Pediatric Surgeons (IAPS); 2 Convener & Secretary, Indian Society for Pediatric Urology

Dear editor,

We read, with great dismay, the editorial on pediatric urology training.[1] We wish to share some concerns about subspecialization in pediatric urology.

We regretfully observe that the tone of the editorial would kill any hope of shaping an emerging specialty - the foundation for training a cadre of surgeons committed to the care of children. Such a foundation can be laid only by a structured training in embryology, neonatal physiology, art of making clinical diagnosis in the absence of subjective history, knack of overcoming narrow margins of safety, delicate tissue handling, pharmacological differences of infants and peculiarities of newborn anatomy. When we consider either urologists or pediatric surgeons subspecializing in pediatric urology, the first question to be answered is, “Who can be easily and effectively trained at the least expense of resources”. Since urologists are not exposed to the principles of pediatric care during their urology training, they have to learn the basics of pediatric care afresh. Consequently, the training will be prolonged with proportionate demand on economic resources. Contrary to this, nearly 10 to 30% of pediatric surgical training is already in pediatric urology.[2,3] Thus, pediatric surgeons are better exposed to urological problems of children than urologists are in the principles of pediatric care. Teaching pediatric surgeons additional skills in renal transplantation and endourology will amount to refining their skills which is not only logical but also easier, effective and economical than to “re-train” adult urologists in pediatric care. That is why in Australia and in many European countries pediatric urology rightfully evolved from pediatric surgery rather than from urology. It is true that in the US general urologists specialize in pediatric urology.[4] Before emulating the American model, one must understand the basic differences between the healthcare systems of India and the US. Contrary to the Indian scenario, US healthcare is insurance-driven. Most of the Indian insurance companies exclude congenital anomalies from reimbursement eligibility. We can seldom match the per capita income, literacy rate, transportation facilities and the structured healthcare delivery system of the US. Copying an American model without having a comparable infrastructure would be a suicidal step in our healthcare system.

“Is exclusive pediatric urology practice commercially viable in India?” is another question to ponder. In Western countries this question can be easily answered using scientific data available on the need of pediatric urology manpower.[5,6] In the absence of such data from India, we can only logically speculate the probability, extrapolating the Western statistics. The birth rate is constantly falling due to effective implementation of “family planning” in our country. The average lifespan of Indians is ever increasing due to the advances in health sciences. As a result of these the Indian demography is steadily shifting towards the geriatric age group [Table 1]. Thus, in future there will be more demand for urologists who can care for geriatric problems such as erectile dysfunctions and cancers rather than those who care for pediatric anomalies.[7] This is further sponsored by the current trend of medical termination of pregnancy based on prenatal diagnosis of fetal anomalies. Consequently, an exclusive pediatric urologist is likely to find progressively diminishing scope in future. Even in the West many urologists prefer to subspecialize in Urogynecology, Andrology and Uro-oncology rather than in pediatric urology.[8,9]

**Table 1:** Projected demographic trends in India

Year	Age under 15	Age 15-64	Age 65 +	Total population
2000	361	604	45	1010
2010	370	747	58	1175
2020	373	882	76	1331

Wikipedia quoting Bhat PNM. Indian Demographic Scenarion 2025. Institute of Economic Growth, New Delhi. Discussion paper No. 27/2001. (Values in millions)

It is obvious that exclusive pediatric urology practice is not commercially viable in most of the Indian centers. Naturally, pediatric urologists have to supplement their income by additional work in other areas. A urologist trained in pediatric urology will, of course, augment his/her income by treating adults as well.[10] Such mixing of adult and pediatric practice cannot be in the best interest of children as it adversely affects the outcome in the latter.[11,12] But, when pediatric surgeons subspecialize in pediatric urology they will supplement their income by treating surgical problems in other organ-systems of children. This is more endurable because “pediatric skills” are maintained without getting diluted with adult practice.

The intention of monopolizing the “pediatric urology market” is too obvious from the editorial. Before any such attempt, proper understanding of the evolutionary background of the specialty of pediatric surgery is mandatory. William Ladd, Robert Gross and Sir Dennis Browne founded this specialty when they realized that surgery in children demanded a dedication and a set of skills that was completely different from adult surgical practice. This applies to urology as much as it applies to other surgical specialties. Specialization based on single-organ-system is appropriate in adults. Contrary to this, congenital and chromosomal anomalies in children frequently involve many organ systems simultaneously.[13] For example, VACTERL anomaly involves anal, vertebral, cardiac, tracheal, esophageal, renal (urological) and limb malformations in the same child. Unlike pediatric surgeons no single 'organ-based specialist' can provide adequate care of all these anomalies. In such a scenario, if multiple specialists are called for, it would merely increase the cost of healthcare without any substantial improvement in the quality of care.[8,13] For an impoverished country like India it would be detrimental. The economic strain is further amplified by the fact that children are not earning members and their parents - who are young and newly married - are yet to secure good position in their careers. Even in the US, Hinman survey[8] expressed similar apprehension about subspecialization in urology.

In the absence of accurate manpower estimation in India, it is unclear as to how the advocates of pediatric urology are going to control the output quantum of trainees. If we overproduce, it may lead to problems including unnecessary surgery, atrophy of skills among practitioners and diminution of major training programs because of lack of patient referrals. If we under-produce, it will be nothing but hypocrisy.

The editorial incriminates that pediatric surgeons hinder the development of pediatric urology in India. To examine the veracity of this stinging indictment, we considered publications as the yardstick. We surveyed the Medline database between 2003 and 2007 (last five years) for publications made by urologists and pediatric surgeons of India. Nine urology journals, three pediatric surgery journals and two pediatric journals [see appendix] that have high impact were considered for sample analysis. With equal fairness, we excluded both the Indian Journal of Urology and the Journal of Indian Association of Pediatric Surgeons as they are still not indexed in Medline. Table 2 summarizes the survey results. It is obvious that pediatric surgeons have made more intellectual contributions in pediatric urology than anyone else. Any accusations on the contrary are, therefore, factitious and *mala fide*. In this context, we wish to remind that it is a group of pediatric surgeons who founded the Indian Society of Pediatric Urology (ISPU) - the country's first academic body of pediatric urology - as early as in 1998. Recently, the Indian Association of Pediatric Surgeons (IAPS) has also formed a subsection of pediatric urology to advance research and training in this area. IAPS and ISPU regularly conduct nationwide courses in pediatric urodynamics and live operative workshops. Indeed, many urology trainees have benefited from them.

**Table 2:** Contribution of ‘Urologists’ versus ‘Pediatric Surgeons’ of India to the science of Pediatric Urology

	Contributions by Indian Urologists	Contributions by Indian Pediatric Surgeons
Total number of articles	201	219
Number of articles on pediatric urology	17 (21%)	64 (79%)

Statistical analysis was done by Chi-square test, *X*^2^ = 29.03; *df* = 1; *P* value < 0.001 (Highly significant)

An ability to perform renal transplantation or endourologic intervention cannot be the defining parameter of a pediatric urologist. In India, the number of children requiring renal transplants is far and few due to many socioeconomic reasons and least because of lack of available skills. Similarly, endourology in children is less about ureteroscopy and Percutaneous Nephrolithotomy (PCNL) and more about safe lower tract work like cystourethroscopy. Fulgurating a newborn urethral valve is far more delicate and demanding than gaining access to the renal pelvis to blast stones. The current generation of pediatric surgeons is adequately trained to treat pediatric urology problems that are common and critical.

As Innes Williams[10] remarked a specialty has both a technical and a social dimension. Ability to do renal transplants, endourology procedures and pediatric urology research are technical aspects of a specialty. They are, no doubt, important. At the same time let us not forget the social dimension of subspecialization. Today, in any medical discipline, specialists are based at metropolitan cities leaving small towns and villages barren of proper healthcare. In these regions surgeons with little or no experience are happily experimenting with children. A good example is a surgeon who does one or two hypospadias repairs every odd year and creates hypospadiac cripples with frightening regularity. Are these not glaring examples of malpractice? Looking into such social discrepancies of urological care is of high priority rather than further splitting medical specialties. Our aim should be to promote the safe practice of pediatric urology in the community and not to indulge in turf wars.

By training adult urologists in pediatric urology we are merely making an attempt to ‘convert’. This will be a half-hearted attempt, entirely uncalled for when a refined and successfully working alternative is already available. In conclusion, we emphasize that pediatric urology is better left to pediatric surgeons who are already doing commendable work in this field. The move of organ-based specialists (who are primarily trained in adults) to take over pediatric subspecialization is perhaps good for them but it is certainly not in the best interest of suffering children.

## Appendix 1: List of Journals Chosen for Sample Analysis

### UROLOGY JOURNALS

1. British Journal of Urology
2. The Journal of Urology
3. Urology
4. European Urology
5. Urologia Internationalis
6. International Urology and Nephrology
7. Scandinavian Journal of Urology and Nephrology
8. International Journal of Urology
9. Urological Research

(Search strategy: These nine journals were searched for author affiliation to an institution in India)

### PEDIATRIC SURGERY JOURNALS

1. Journal of Pediatric Surgery
2. Pediatric Surgery International
3. European Journal of Pediatric Surgery

(Search strategy: These three journals were searched for author affiliation to an institution in India)

### PEDIATRIC SURGERY JOURNALS

1. Indian Pediatrics
2. Indian Journal of Pediatrics

(Search strategy: These two journals were searched for author affiliation to urology or pediatric surgery departments and for authors' affiliation to an institution in India)
